# Identification of novel non-*HFE* mutations in Chinese patients with hereditary hemochromatosis

**DOI:** 10.1186/s13023-022-02349-y

**Published:** 2022-06-06

**Authors:** Wei Zhang, Yanmeng Li, Anjian Xu, Qin Ouyang, Liyan Wu, Donghu Zhou, Lina Wu, Bei Zhang, Xinyan Zhao, Yu Wang, Xiaoming Wang, Weijia Duan, Qianyi Wang, Hong You, Jian Huang, Xiaojuan Ou, Jidong Jia, China Registry of Genetic/Metabolic Liver Diseases (CR-GMLD) Group

**Affiliations:** 1 Liver Research Center, Beijing Friendship Hospital, Capital Medical University; Beijing Key Laboratory of Translational Medicine on Liver Cirrhosis, 95 Yong-An Road, Beijing, 100050 China; 2 National Clinical Research Center for Digestive Diseases, Beijing, 100050 China; 3 Experimental Center, Beijing Friendship Hospital, Capital Medical University, Beijing, 100050 China

**Keywords:** Hereditary hemochromatosis, Non-*HFE*, *UBE2O*, *PCSK7*, Gene mutation, Iron overload

## Abstract

**Backgrounds:**

Hereditary hemochromatosis (HH) is mainly caused by homozygous p.C282Y mutations in *HFE* in the Caucasians. We recently reported non-*HFE* mutations constitute the major cause of HH in Chinese. However, there is still a relatively high proportion of cases with primary iron overload from unexplained causes. We aimed to explore novel non-*HFE* mutations in Chinese patients with primary iron overload.

**Methods:**

Whole exome sequence was conducted to screen mutations in novel HH-related genes in the 9 cases with unexplained primary iron overload. Then the representative candidate genes were screened for mutations in another cohort of 18 HH cases. The biological function of the selected genes and variants were analyzed in vitro.

**Results:**

Whole exome sequencing of 9 cases with unexplained primary iron overload identified 42 missense variants in 40 genes associated with iron metabolism pathway genes such as *UBE2O* p.K689R and *PCSK7* p.R711W. Subsequent Sanger sequencing of the *UBE2O* and *PCSK7* genes in the 27 cases with primary iron overload identified p.K689R in *UBE2O*, p.R711W and p.V143F in *PCSK7* at frequency of 2/27,1/27 and 2/27 respectively. In vitro siRNA interference of *UBE2O* and *PCSK7* resulted in down-regulated *HAMP* mRNA expression. Adenovirus generation of *UBE2O* p.K689R in cell lines resulted in increased expression of SMAD6 and SMAD7 and downregulation of p-SMAD1/5 and *HAMP* expression, and the reduction of hepcidin level.

**Conclusions:**

Our study identified a series of novel candidate non-*HFE* mutations in Chinese patients with HH. These may provide insights into the genetic basis of unexplained primary iron overload.

**Supplementary Information:**

The online version contains supplementary material available at 10.1186/s13023-022-02349-y.

## Introduction

Hereditary hemochromatosis (HH) is characterized by excessive iron deposition in the liver and other organs including heart, pancreas, bone and joints, and pituitary gland, leading to hepatic and extra-hepatic complications. This disorder is related to deficiency or resistance of hepcidin, which is produced by hepatocytes in the liver and blocks the entry of iron into the plasma by inducing the internalization and degradation of the iron exporter ferroportin (FPN1) [1]. The known causes of HH include gene mutations in upstream regulators of the expression of hepcidin, such as human hemochromatosis protein (*HFE)*, hemojuvelin (*HJV*)*,* hepcidin gene (*HAMP*)*,* transferrin receptor-2 (*TFR2*), and its target *FPN1* (also known as *SLC40A1*) [2]*.*

In Caucasians patients with HH *HFE* p.C282Y is the most common mutation, accounting for more than 90% of cases. Non-*HFE*-related HH accounts for a minority of the remaining cases. However, some patients with primary iron overload do not show mutations in the aforementioned genes. Studies with next generation whole exome sequencing (NGWES) found that mutations in the pro-peptide domain region of *BMP6* and mutations in *NMBR* were new genetic factors predisposing to primary iron overload [3, 4].

In Chinese HH patients the gene mutations are quite different from those in Caucasian patients [5, 6]. In China, the prevalence of HH is very rare, whereas several mutations in non-*HFE* genes including *HJV* p.Q312X, p.C321X [7, 8] and p.I281T [8], *TFR2* p.I238M and G430R [9], and *SLC40A1* p.W158C [10], p.S209L [10] were identified in primary iron overload cases. Our group have identified recently several mutations in non-*HFE* genes in HH, including *HJV* p.E3D, p.H104R, and p.V274M, *TFR2* p.A302E and p.L745R, and *SLC40A1* p.Y333H [5, 11]. We further identified other novel genetic variants in genes involved in iron metabolism, such as *TMPRSS6* p.T331M, *BMP4* p.R269Q, *SUGP2* p.R639Q and *DENND3* p.L708V [5], and specifically we identified the first HH case with compound heterozygosity p.C282Y/p.R71X in *HFE* [12].

However, there are still some cases with primary iron overload had undefined genetic causes (approximately 30% of patients in the China Registry of Genetic/Metabolic Liver Diseases; data not shown). Therefore, in the present study, we explored novel HH-related genes by whole exome sequencing (WES) in a cohort of cases with primary iron overload with unexplained causes. We first identified mutations in a series of potential iron metabolism–related genes such as ubiquitin-conjugating enzyme E2 O (*UBE2O*), proprotein convertase subtilisin/kexin type 7 (*PCSK7*), then we analyzed the function and explored the mechanisms of representative genes and variants.

## Patients and methods

### Patients

Patients with primary iron overload were enrolled at the China Registry of Genetic/Metabolic Liver Diseases for the genetic analysis of mutations in HH-related genes.

The diagnosis of HH was based on the American Association for the Study of Liver Diseases 2011 practice guidelines on hemochromatosis[13] as follows: (1) transferrin saturation (TS) ≥ 45% and/or elevated ferritin (> 300 ng/mL in men and postmenopausal women or > 200 ng/mL in premenopausal women); (2) iron overload in the liver and/or spleen on magnetic resonance imaging of the liver or liver histology; and (3) excluded causes of secondary iron overload, such as alcoholic or other chronic liver disease, iron-overloading anemia, and parenteral iron overload.

This study was approved by the Clinical Research Ethics Committee of Beijing Friendship Hospital, Capital Medical University (No. 2016-P2-061-01). Informed and written consent was obtained from all patients.

### Blood sample collection and DNA extraction

Genomic DNA was extracted from whole blood using a Genomic DNA Purification Kit (Qiagen, Valencia, CA, USA). Quality control was performed by evaluating the 260/280 nM absorbance ratio and gel electrophoresis.

### Identification of novel mutations in the discovery cohort by WES

WES was performed on DNA extracted from the peripheral blood of nine unrelated patients with primary iron overload, in which mutations in known HH-related genes (*HFE, HJV, HAMP, TFR2, SLC40A1*) could not explain the severity of iron overload. Table 1 summarizes the clinical features of these patients.

**Table 1:** Clinical Characteristics of discovery cohort with primary iron overload

	Age	Sex	SF (ng/ml)	TS (%)	AST (U/L) (15–40)	ALT (U/L) (9–50)	T-Bil (umol/L)	D-Bil (umol/L)	γ-GGT (U/L) (8–55)	Iron overload on MRI	Iron overload on liver biopsy	End-organ manifestations
D1	62	F	5346	99	49	54	9	2	45	Liver, spleen and pancreas	Predominant in hepatocytes	Abnormal liver function test
D2	28	M	737	46	35	40	192	159	30	ND	Predominant in hepatocytes	Jaundice
D3	38	F	843	96	53	30	40	14		Liver and spleen	Predominant in hepatocytes	Skin pigmentation, liver cirrhosis, diabetes, amenorrhea
D4	31	M	2013	98	96	69	96	31	73	Liver, spleen and pancreas	ND	Liver cirrhosis
D5	46	M	2000	85	109	215	32	15	291	Liver, spleen	ND	Abnormal liver function test
D6	45	M	685	ND	21	16	72	10	18	Liver	Predominant in hepatocytes	Abnormal liver function test
D7	29	M	418	93	28	20	51	17	17	ND	Predominant in hepatocytes	Skin pigmentation, liver cirrhosis
D8	31	M	2000	93	154	452	–	–	99	ND	Predominant in Kupffer cells	Abnormal liver function test
D9	74	M	436	71	30	20	34	8	100	Liver	ND	Liver cirrhosis, atrial fibrillation

ALT, alanine aminotransferase; AST, aspartate aminotransferase; ND: Not done; SF, serum ferritin; T-Bil, total bilirubin; D-Bil, direct bilirubin; TS, transferrin saturation; γ‐GGT, gamma glutamyl transpeptidase; MRI, magnetic resonance imaging

A targeted exome library with an insert size of 150–200 bp was constructed from approximately 1 µg of genomic DNA by an exome capture strategy using a GenCap custom exome enrichment kit (MyGenostics, Beijing, China). The Illumina HiSeq 2000 platform was used to generate paired-end 100 bp raw reads from each enriched library according to the manufacturer’s protocol. The 100 bp paired-end reads were aligned against NCBI build 37 of the human genome using Burrows Wheeler Aligner. Duplicate reads were marked, local indel realignment performed and base quality scores were recalibrated for each sample with the Genome Analysis Toolkit.

Novel point mutations were identified using MuTect, while indel variants were identified using Somatic Indel Detector in the Genome Analysis Toolkit. The potential pathogenic variants were confirmed by Sanger sequencing.

The criteria for the screening of the mutations in potential iron metabolism–related genes were as follow: in public data bases, the population frequency is less than 1%, no report or prediction as benign or likely benign, and predicted as disease causing by at least one of the prediction tools SIFT, Polyphen-2 and Mutation Taster.

### Screening for the newly discovered gene mutations in a cohort of primary iron overload by Sanger sequencing

To investigate the novel candidate genes, we enrolled 18 unrelated patients with HH as the validation cohort. Among the 18 patients, 3 carried *HFE* mutations, 7 carried *HJV* mutations, 3 carried *TFR2* mutations, 7 carried *SLC40A1* mutations, 2 carried *SUGP2* mutations, 1 patient carried *TMPRSS6* mutation, and 1 patient carried *BMP4* mutation. The clinical features of these 18 cases are shown in Additional file 1: Table S1.

All exons of *UBE2O* and *PCSK7* were PCR-amplified with associated boundary regions using specific primers (see Additional file 2: Table S2). PCR amplification was performed in an ABI Veriti 96 PCR cycler (Applied Biosystems, Foster City, CA, USA). PCR products were sequenced using an automated ABI 3730 DNA sequencer (Applied Biosystems).

## Fuctional analysis of the newly discovered gene mutations

### Cell culture and transfection

The human hepatocellular carcinoma (HCC) cell lines Huh-7 and HepG2 were obtained from the Cell Resource Center of the Chinese Academy of Medical Science (Beijing, China). Huh-7 and HepG2 cells were cultured as described previously [5, 14].

For the siRNA-mediated transient knockdown of gene expression, Huh-7 and HepG2 cells (5 × 10^5^ cells) were transfected with 20 nM siRNA using LTX reagent (Invitrogen, USA) in accordance with the manufacturer’s instructions.

For the adenovirus generation and establishment of stable UBE2O-overexpressing and UBE2O-K689R cell lines, Huh-7 and HepG2 cells were infected with the adenovirus. After 24 h, we confirmed infection by observed expression of red fluorescence protein. After 24 h, assays were performed.

### siRNA interference of UBE2O and PCSK7

Gene knockdown was performed using *UBE2O* siRNAs (ID siG000063893A and ID siG000063893B), *PCSK7* siRNA (ID siG000009159), and the negative control siRNA (ID siN0000001-1–5) (RiboBio, Guangzhou, China).

### Adenovirus generation for UBE2O and UBE2O-K689R expression

The *UBE2O* and *UBE2O* p.K689R sequences were cloned into the pADV-EF1-mScarlet-CMV-MCS-3xFLAG vector, and Huh-7 and HepG2 cells were infected with the adenovirus.

### Real-time PCR

The isolation of total RNA from cell lines and real-time PCR assays were conducted as described previously [11]. The primer sequences are listed in Additional file 2: Table S2. GAPDH mRNA served as control.

### Immunofluorescence staining

Immunofluorescence analysis was conducted as described previously [5, 14]. Cells were incubated with a primary antibody directed against rabbit anti-hepcidin (1:50; Abcam, USA) at 4 °C overnight. After three 5 min washes with phosphate-buffered saline (PBS), cells were incubated with anti-rabbit Alex 488-conjugated secondary antibodies (1:500; Invitrogen) for 1 h at room temperature. After additional PBS washes, cells were mounted on a slide in mounting medium (Molecular Probes). Cells were then examined and photographed using an FV 300 confocal microscope (Olympus, Tokyo, Japan).

### Western blotting

Western blot analysis was performed as described previously [5, 14]. Membranes were incubated with rabbit anti-UBE2O (1:1000; Novus), rabbit anti-Smad6 (1:1000; BD), rabbit anti-p-Smad1/5 (1:1000; Sigma), rabbit anti-tSmad1 (1:1000; Cell signaling technology), mouse anti-Smad7 (1:100; RD) or mouse anti-GAPDH antibodies (1:5000; Zhongshanjinqiao) overnight at 4 °C, followed by incubation with horseradish peroxidase–conjugated goat anti-mouse or anti-rabbit antibodies (1:5000; Zhongshanjinqiao) for 1 h at room temperature. Target proteins were detected using Immobilon Western Chemiluminescent HRP Substrate (Millipore, Billerica, MA, USA).

### Enzyme-linked immunosorbent assay (ELISA)

The human hepcidin detection kit (Cloud-Clone Corp, SEB979Hu) was used to determine the concentration of hepcidin in cell culture supernatants according to the manufacturer’s protocol.

### Statistical analysis

All experiments were carried out at least three times. The Fisher’s exact test was used to determine the difference of mutation frequency between patients and healthy subjects using SPSS v21.0 software. *P* < 0.05 was considered to indicate statistical significance.

## Results

### Identification of novel non-HFE variants in primary iron-overload cases by WES

**Fig. 1:**
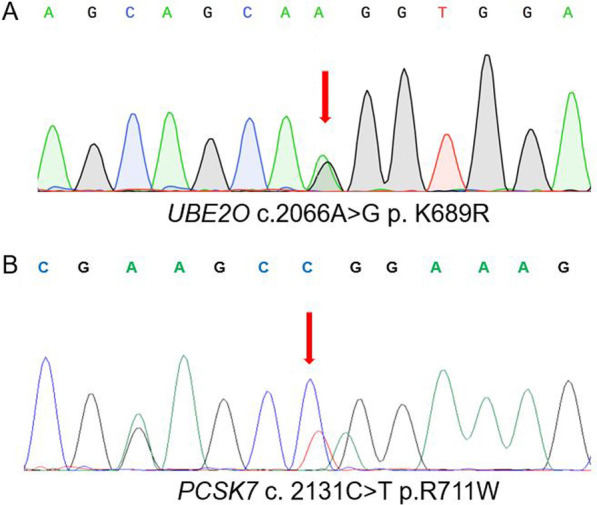
Representative sequencing of the novel variants in *UBE2O*, and *PCSK7* in cases with primary iron overload. Sequencing of the heterozygous missense mutations *UBE2O* p.K689R (**A**) and *PCSK7* p.R711W (**B**)

We performed WES in 9 unrelated patients with unexplained primary iron overload. Based on the criteria as described in the methods, we identified a total of 69 mutations in 61 genes associated with iron metabolism pathway (Additional file 3: Table S3, Additional file 4), including 42 missense variants in 40 genes, such as *UBE2O* p.K689R, *PCSK7* p.R711W (Fig. 1A, B, Table 2, Additional file 5: Table S4). In addition, 116 unknown potential iron metabolism–related genes were identified with mutations in at least two of the nine cases, such as *COL6A5*, *MRRF* and *MUC5B* genes (Additional file 6).

**Table 2:** *UBE2O* and *PCSK7* missense mutations identified in patients with primary iron overload

Gene (accession number)	Amino acid change	Base change	ExAC	gnomAD_exome	Polyphen-2		SIFT		Mutation Taster		Frequency of mutation detected in primary iron overload
					Prediction	Score	Prediction	Score	Prediction	Score	
*UBE2O* (NM_022066)	p.K689R	c.2066A > G	0.0002	0.0002	Probably damaging	0.993	Tolerable	0.853	Disease causing	1.000	2/27
*PCSK7* (NM_004716)	p.R711W	c.2131C > T	0.0011	0.0012	Probably damaging	0.978	Damaging	0.005	Polymorphism	1.000	1/27
	p.V143F	c.427 G > T	0.0021	0.0020	Possibly damaging	0.528	Tolerable	0.262	Polymorphism	0.997	2/27

### Screening of UBE2O and PCSK7 variants in patients with primary iron overload cohort by Sanger sequencing

We next performed Sanger sequencing for the two identified genes in 27 primary iron-overload cases. Two cases carried *UBE2O* p.K689R (7.4%, 2/27), one case carried p. R711W (3.7%, 1/27) and two cases carried p. V143F (7.4%, 2/27) in *PCSK7*, and with allele frequency of 0.0002, 0.0012, 0.0020 in gnomAD database (Table 2).

### HAMP expression was decreased in UBE2O- and PCSK7 -knockdown HCC cells

**Fig. 2:**
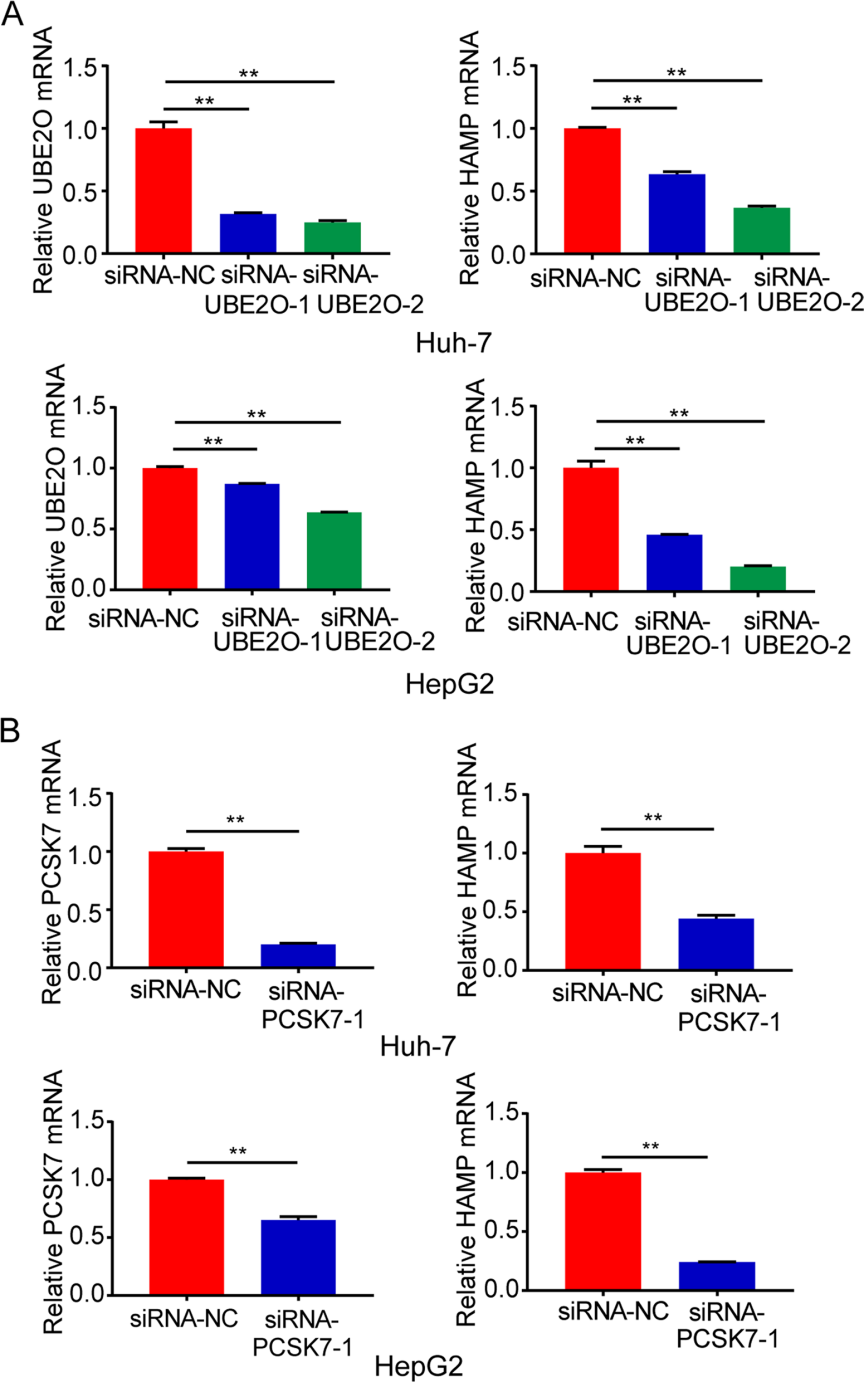
Analysis of *HAMP* mRNA expression in *UBE2O* and *PCSK7* knockdown HCC cells. **A** *HAMP* mRNA levels in Huh-7 and HepG2 cells transfected with *UBE2O* siRNA or control siRNA. **B** *HAMP* mRNA levels in Huh-7 and HepG2 cells transfected with *PCSK7* siRNA or control siRNA

We next conducted in vitro siRNA interference of representative genes *UBE2O*, *PCSK7* to analyze their effects on *HAMP* mRNA expression. Results from qRT-PCR showed that the level of *HAMP* mRNA, which encodes hepcidin, was decreased in UBE2O- and PCSK7-knockdown HCC cells compared with control cells (Fig. 2A, B). These results indicate that knockdown of *UBE2O*, or *PCSK7* gene reduces the expression of *HAMP* mRNA.

### The function of Smad6 and Smad7 enhanced in UBE2O-knockdown HCC cells

**Fig. 3:**
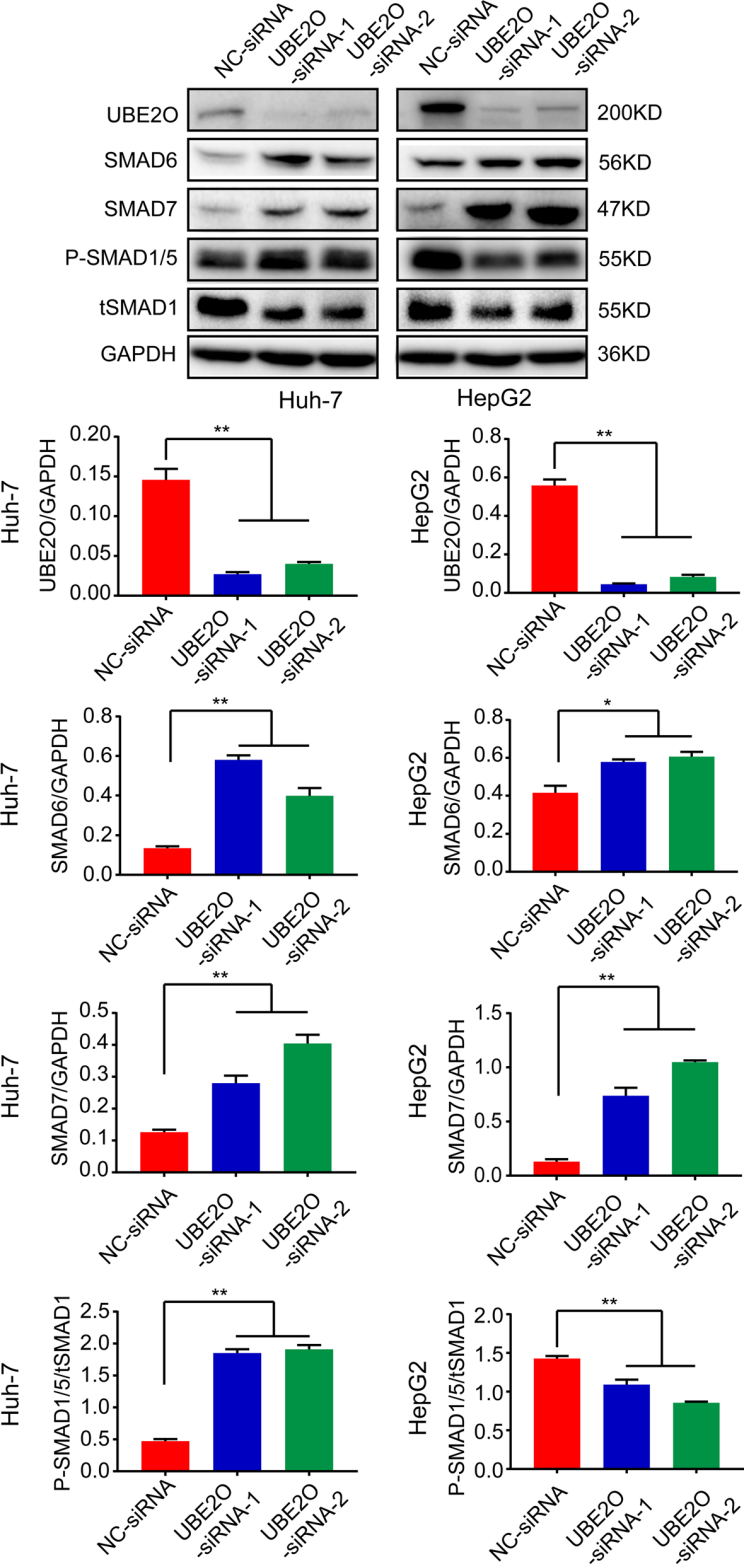
Analysis of SMAD6 and SMAD7 expression in *UBE2O* knockdown HCC cells. Western blot analysis of SMAD6, SMAD7, and pSmad1/5 expression in *UBE2O* knockdown Huh-7 and HepG2 cells showed that higher expression of Smad6 and Smad7 in UBE2O-knockdown HCC cells, and higher ratio of pSmad1/5/tSmad1 in Huh-7 cells, but lower ratio of pSmad1/5/tSmad1 in HepG2 cells

We further analyzed the expression of I-SMADs (SMAD6 and SMAD7) by Western blot. Higher expression of Smad6 and Smad7 was observed in UBE2O-knockdown HCC cells. Moreover, we found higher ratio of pSmad1/5/tSmad1 in Huh-7 cells, but lower ratio of pSmad1/5/tSmad1 in HepG2 cells (Fig. 3). The results indicate the inhibition of Smad6 and Smad7 on pSmad1/5 level in UBE2O-knockdown HepG2 cells. However, this effect was not observed in Huh-7 cells (Fig. 3).

### The inhibition of hepcidin expression by UBE2O p.K689R was through inhibiting Smad1/5 phosphorylation by enhancing the function of Smad6 and Smad7

**Fig. 4:**
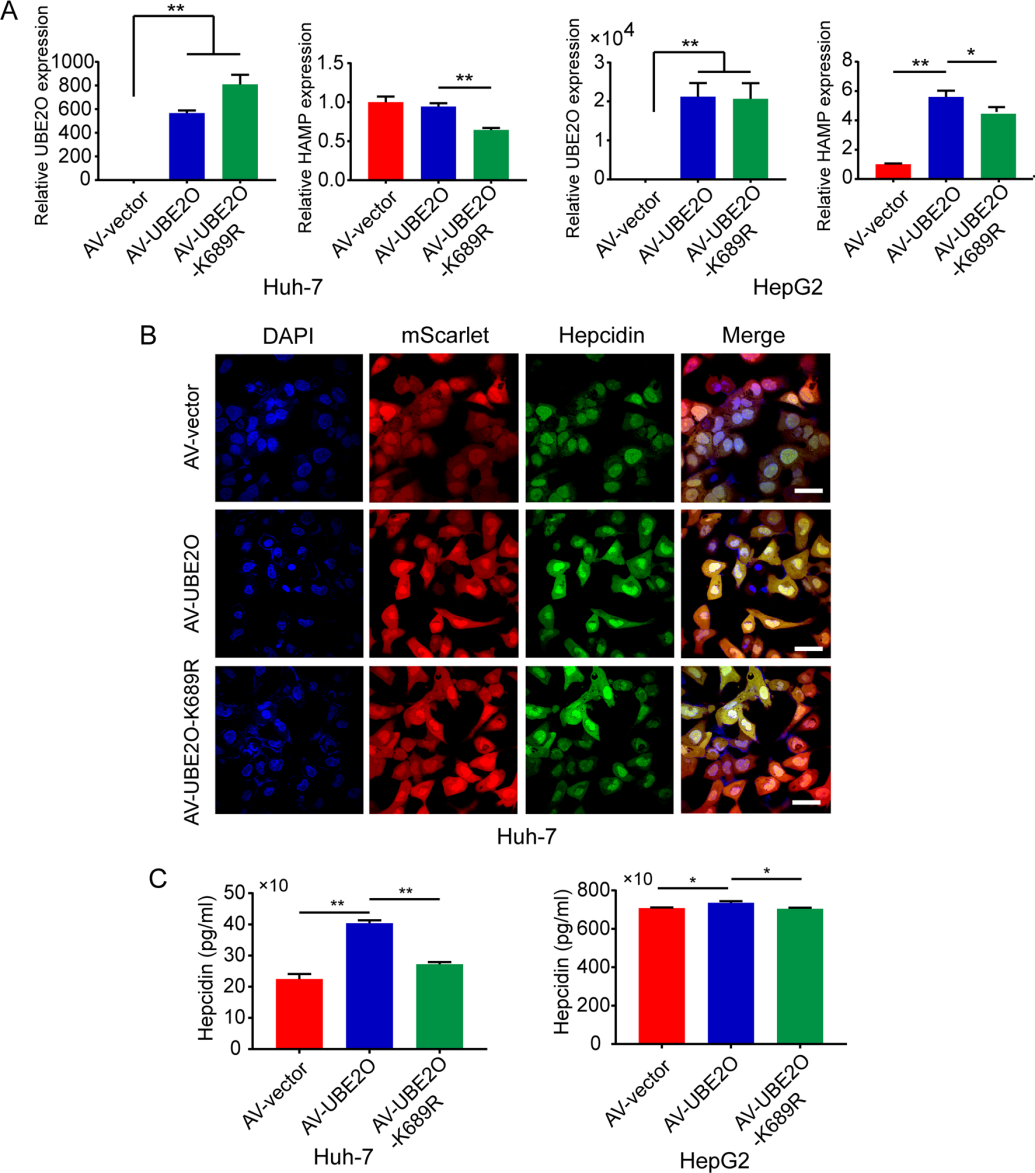
Analysis of HAMP expression in Huh7 and HepG2 cells infected with *UBE2O* or *UBE2O* p.K689R adenovirus. **A**. *HAMP* mRNA levels in Huh-7 and HepG2 cells were analyzed by real-time PCR assays. **B** and **C** Hepcidin was analyzed in Huh-7 and HepG2 cells by immunofluorescence and ELISA

It has been known UBE2O monoubiquitinates inhibitory-Smads (Smad6 and Smad7), blocking its association with activated type I receptor BMPRI and resulting in increased BMP/SMAD signaling [15]. We used *UBE2O* p.K689R as a representative for initial mechanism study. Results from qRT-PCR showed that mutant UBE2O (p.K689R) down-regulated *HAMP* mRNA level to a greater extent than wild-type UBE2O in HepG2 cells and Huh-7 cells (Fig. 4A). Consistent with the reduced expression of *HAMP* mRNA caused by UBE2O p.K689R, the hepcidin level in cells expressing mutant UBE2O was lower than cells expressing wild-type UBE2O in ELISA and immunofluorescence staining assays (Fig. 4B, C).

**Fig. 5:**
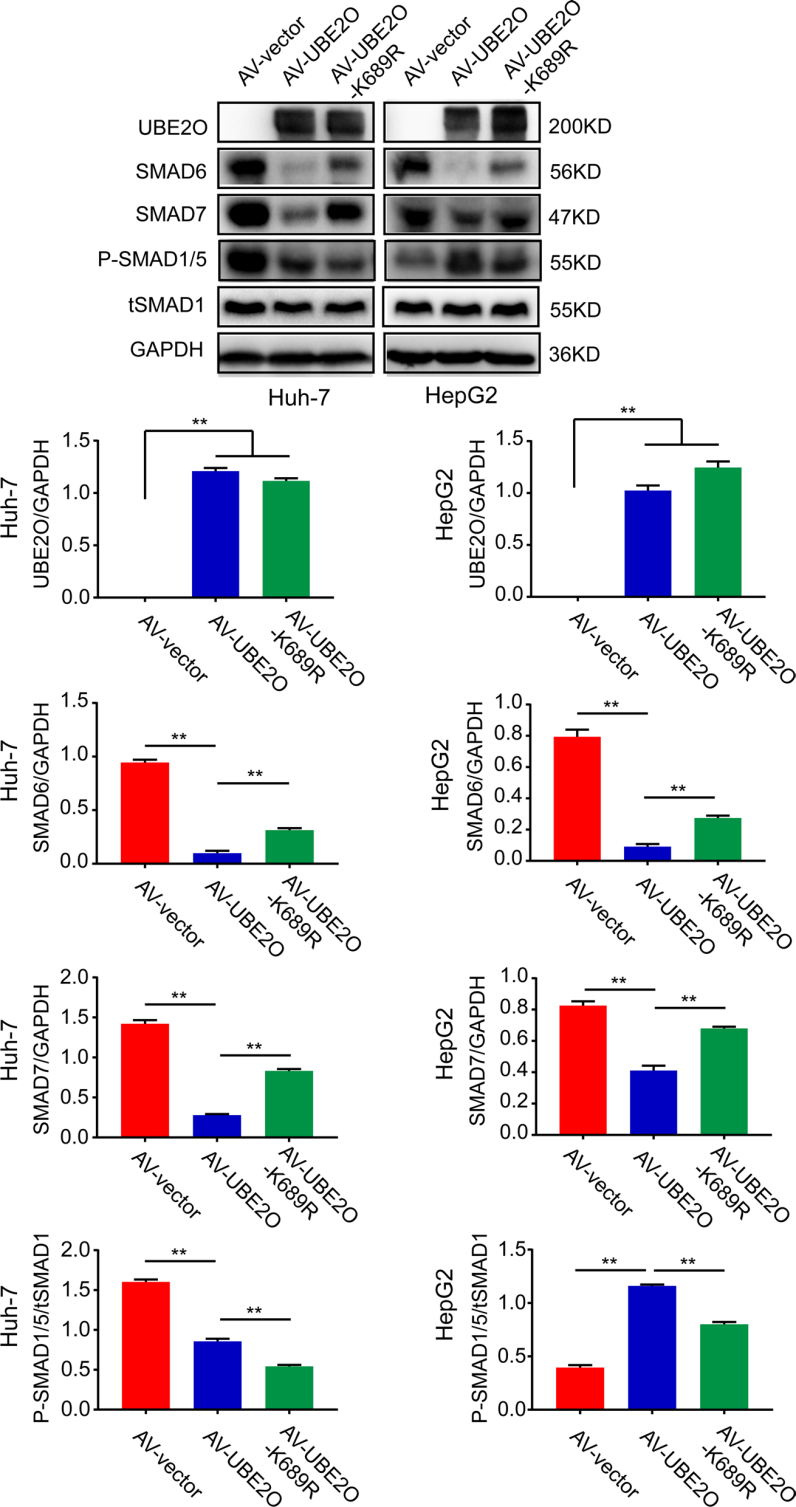
Analysis of SMAD6 and SMAD7 expression in Huh7 and HepG2 cells infected with *UBE2O* or *UBE2O* p.K689R adenovirus. Western blot analysis of SMAD6, SMAD7, and pSmad1/5 expression in Huh-7 and HepG2 cells showed higher expression of Smad6 and Smad7 and lower ratio of pSmad1/5/tSmad1 in *UBE2O* p.K689R HCC cells than wild-type HCC cells

Western blot analysis revealed higher expression of Smad6 and Smad7 and lower ratio of pSmad1/5/tSmad1 in *UBE2O* p.K689R HCC cells than wild-type HCC cells (Fig. 5). These results indicate that the inhibition of hepcidin expression by UBE2O p.K689R may be through inhibiting ubiquitination-mediated degradation of Smad6 and Smad7, and the subsequent inhibition of SMAD1 phosphorylation.

## Discussion

In the present study, we firstly identified novel mutations by NGWES in a small number of patients with unexplained primary iron overload*,* then we screened for the mutations in the representative newly identified genes, *UBE2O* and *PCSK7,* in a larger cohort of primary iron overload patients. We found recurrent p.K689R variant in *UBE2O* and a high frequency of mutations in *PCSK7* in primary iron overload patients. Functional studies indicated that mutation in *UBE2O* and *PCSK7* may play a role in the regulation of *HAMP*/hepcidin expression. Taken together, our study identified a series of novel candidate non-*HFE* mutations in Chinese patients with HH, and as the representative candidate, the *UBE2O* and *PCSK7* may function in iron metabolism, which is essential for the HH cases that are difficult to interpret.

UBE2O (UniProt accession #Q9C0C9) is encoded at the 17q25 region. As an E2/E3 hybrid ubiquitin-protein ligase [16], UBE2O displays roles of an E2 ubiquitin conjugating enzyme and E3 ubiquitin ligase. UBE2O contains three conserved regions (CR1, CR2, and CR3), a coiled-coil domain, a UBC domain, and two putative nuclear localization sequences [17]. UBE2O acts on a broad spectrum of targets and execute multiple biological functions. For example, UBE2O negatively regulates TRAF6-mediated NF-κB activation by inhibiting TRAF6 polyubiquitination [18]. UBE2O multi-monoubiquitinates the nuclear localization signal of BAP1, inducing its cytoplasmic sequestration [17]. UBE2O promotes the proliferation, epithelial-mesenchymal transformation, and stemness properties of breast cancer cells through the UBE2O/AMPKα2/mTORC1 positive feedback loop [19], and UBE2O facilitates tumorigenesis and radioresistance by promoting Mxi1 ubiquitination and degradation [20]. Thus, UBE2O targets several proteins for ubiquitination and has been implicated in chromatin-associated protein nuclear transport, adipogenesis, tumor progression, and metastasis [16, 18–22]. However, no study has linked UBE2O to the regulation of iron metabolism. In the present study, we found that two HH patients carried p.K689R in *UBE2O*. However, the mechanism of UBE2O regulating iron metabolism is not clear.

Hepcidin deficiency is the common feature in HH and is responsible for iron overload in HH. Hepcidin acts in close connection with ferroportin to regulate iron metabolism. When plasma or hepatocyte iron concentrations increase, signaling pathways including ERK/MAPK and BMP/SMAD pathways are activated and induce hepcidin mRNA expression, leading to increased plasma hepcidin. Hepcidin then interacts with ferroportin, resulting in decreased duodenal iron absorption and a decreased release of iron from the spleen coming from erythrophagocytosis [1]. The BMP/SMAD pathway is the major pathway for transcriptional regulation of hepcidin expression in hepatocytes. SMAD6 and SMAD7 are inhibitory SMADs that are induced by BMP/SMAD signaling and inhibit the BMP/SMAD pathway by interfering with type I receptor function or SMAD complex formation [2]. Zhang and colleagues found that UBE2O monoubiquitinates SMAD6, blocking its association with activated type I receptor and resulting in increased BMP/SMAD signaling [15]. Furthermore, the authors showed that forced UBE2O expression in C3H10T1/2 cells potentiated BMP7-induced SMAD1 phosphorylation and adipocyte differentiation, and forced UBE2O expression in C2C12 cells enhanced BMP6-induced SMAD1 phosphorylation and osteoblast differentiation [15]. This indicates that UBE2O may regulate the BMP/SMAD pathway through the ubiquitination of I-SMADs.

In the present study, we found increased expression of Smad6 and Smad7 and decreased expression of HAMP mRNA in *UBE2O*-knockdown cells, and the level of p-SMAD1/5 decreased in *UBE2O*-knockdown HepG2 cells. However, the decreased p-SMAD1/5 level was not observed in *UBE2O*-knockdown Huh7 cells. Previous studies have shown that the expression of p-SMAD1/5 is always inconsistent with that of HAMP or hepcidin in certain cell types. Specifically, the level of hepcidin could be affected by a variety of factors in addition to p-SMAD1/5, such as inflammation and stress. In *UBE2O* p.K689R–expressing cells, the expression of SMAD6 and SMAD7 increased and the expression of p-SMAD1/5 decreased, along with reduced expression of HAMP mRNA. This suggests that UBE2O may influence iron metabolism by regulating the BMP/SMAD pathway. We speculate that UBE2O regulates the BMP/SMAD pathway through ubiquitination of SMAD6 and SMAD7, and we will explore this hypothesis in future research.

We also identified high frequency of *PCSK7* variations in patients with primary iron overload. PCSK7 is a family member of nine secretory serine proteases related to bacterial subtilisin and yeast kexin (*PCSK1*–*PCSK9*) [23]. Oexle et al. established a strong link between plasma levels of the soluble human transferrin receptor 1 and *PCSK7* by a genome-wide association study [24]. Guillemot et al. later found that PCSK7 acted in iron homeostasis by directly shedding hTfR1 by cleavage at an atypical site and showed that furin alone activates hepcidin [25]. Our study revealed that *HAMP* mRNA was decreased in *PCSK7*-knockdown HCC cells, further indicating that *PCSK7* may be a candidate gene or modifier gene causing iron overload involved in HH.

This study has several limitations. First, this study lacked in vivo data, and animal models are needed to clarify the effect of UBE2O on iron metabolism. Second, the mechanisms by which PCSK7 impact hepcidin expression are unknown, and thus the molecular signaling involving hepcidin regulation by *PCSK7* needs further study. Finally, the genetic association needs further validation in a larger cohort of patients with primary iron overload.

In conclusion, our study identified a series of novel candidate non-*HFE* mutations in Chinese patients with HH. which may provide insights into the genetic basis of the unexplained primary iron overload.

## Supplementary Information

**Additional file 1**.** Table S1**. Clinical characteristics of validation cohort with primary iron overload.

**Additional file 2**.** Table S2**. Primers for the Sanger sequencing of *UBE2O*, *PCSK-7* gene.

**Additional file 3**.** Table S3**. Quality control of the whole exome sequence of the 9 cases with primary iron overload.

**Additional file 4**. Mutations identified in known iron metabolism–related genes or genes associated with iron metabolism pathway by NGWES.

**Additional file 5**.** Table S4**. Missense variants in genes associated with iron metabolism pathway identified in patients with primary iron overload by NGWES.

**Additional file 6**. Unknown potential iron metabolism–related genes identified with mutations in at least two of the nine cases by NGWES.

## Data Availability

The datasets used and/or analyzed during the current study are available from the corresponding author on reasonable request.

## Abbreviations

HH: Hereditary hemochromatosis
*HFE*: Human hemochromatosis protein
*HJV*: Hemojuvelin
*HAMP*: Hepcidin antimicrobial peptide
*SLC40A1*: Solute carrier family 40 member 1
FPN1: Ferroportin 1
BMP: Bone morphogenetic protein
SMAD: Small mothers against decapentaplegic
*UBE2O*: Ubiquitin-conjugating enzyme E2O
*PCSK**7*: Proprotein convertase subtilisin/kexin type 7
TS: Transferrin saturation
PBS: Phosphate-buffered saline
NGWES: Next generation whole exome sequencing
