# Supplementary material for: Identification of novel non-HFE mutations in Chinese patients with hereditary hemochromatosis
Source: Orphanet J Rare Dis. 2022 Jun 6;17:216. doi: 10.1186/s13023-022-02349-y (PMC9169345; doi:10.1186/s13023-022-02349-y)
Supplement: Supplementary file 1 — Additional file 1. Table S1. Clinical characteristics of validation cohort with primary iron overload. [file 13023_2022_2349_MOESM1_ESM.docx]

Table S1. Clinical Characteristics of validation cohort with primary iron overload

| No | Mutation in HH related genes | Age | Sex | SF (ng/ml) | TS  (%) | AST  (U/L)  (15-40) | ALT  (U/L)  (9-50) | T-Bil  (umol/L) | D-Bil  (umol/L) | γ-GGT  (U/L)  (8-55) | Iron overload on MRI | Iron overload on liver biopsy | End-organ manifestations |
| --- | --- | --- | --- | --- | --- | --- | --- | --- | --- | --- | --- | --- | --- |
| 1 | *HJV* Q312X（Homo） | 22 | F | 2995 | 89.1 | 98 | 118 | 15.7 | 3.7 | 27 | Liver and pancreas | Predominant in hepatocytes | Lethargy, skin pigmentation, amenorrhea, abnormal liver function test |
| 2 | *SLC40A1* IVS3+10delGTT | 48 | F | 7078 | 99.6 | 68 | 62 | 7.4 | 0.9 | 49 | Liver and spleen | Predominant in hepatocytes | Diabetes, amenorrhea and abnormal liver function test |
| 3 | *HJV* p. Q6H+C321X+H104R，*TFR2* p. A75V | 22 | M | 6678 | 100 | 62.8 | 27 | 33.2 | 6.8 | 42 | ND | Predominant in hepatocytes | Abnormal liver function test |
| 4 | *SUGP2* p. R639Q，  TMPRSS6 p. T331M | 36 | M | 420 | 84.1 | 57.2 | 73 | 16.8 | 3.7 | 202 | Liver | ND | Abnormal liver function test |
| 5 | *HJV* p.E3D，*BMP4* p. R269Q，*SUGP2* p. R639Q | 53 | F | 1402 | 49 | 144 | 232 | 123 | 17 | 14 | Liver and pancreas | Predominant in hepatocytes | Lethargy, jaundice, abnormal liver function test |
| 6 | *HJV* p. Q6H+C321X+V274M | 57 | M | 4001 | 93 | 127 | 118 | 25 | 11 | 716 | Liver | Predominant in hepatocytes | Abnormal liver function test |
| 7 | *HJV* p. F103L（Homo） | 36 | F | 2000 | 96 | 86 | 104 | 13 | 3 | 35 | Liver | Predominant in hepatocytes | Abnormal liver function test, amenorrhea |
| 8 | *SLC40A1* p. Y333H | 49 | M | 7445 | 97 | 83 | 87 | 12 | 2 | 345 | Liver and spleen | ND | Liver cirrhosis |
| 9 | *SLC40A1* p. Y333H | 60 | M | 15000 | 94 | 198 | 304 | 29 | 5 | 256 | Liver, spleen, and pancreas | Predominant in hepatocytes | Liver cirrhosis, diabetes, skin pigmentation |
| 10 | *SLC40A1* p. N144D, *TFR2* R336H | 57 | F | 5886 | 71 | 64 | 73 | 12 | 2 | 24 | Liver, spleen, and pancreas | Predominant in hepatocytes | Liver fibrosis and amenorrhea |
| 11 | *SLC40A1* p. Y333H, *TFR2*  IVS287-30A>G, IVS1768-21G>T | 66 | F | 1446 | 93 | 39 | 20 | 35 | 9 | 21 | Liver, spleen, and pancreas | ND | Liver cirrhosis, Diabetes and skin pigmentation |
| 12 | - | 63 | M | 2497 | 98 | 33 | 45 | 8.1 | 1.3 | 23 | Liver and spleen | Predominant in hepatocytes | Abnormal liver function |
| 13 | *HFE* p. C282Y/R71X | 28 | M | 2153 | 92 | 47 | 80 | 9 | 2 | 23 | Liver | Predominant in hepatocytes | Abnormal liver function test |
| 14 | *SLC40A1* p. V511I，*HFE*  p. Q21R | 48 | M | 2267 | 92 | 57 | 84 | 16 | 3 | 52 | Liver, spleen, and pancreas | Predominant in hepatocytes | Abnormal liver function test and diabetes |
| 15 | *SLC40A1* p.V221V(homo) | 30 | M | 597 | ND | 59 | 43 | 171 | 91 | 15 | ND | Predominant in hepatocytes | Liver cirrhosis |
| 16 | *HFE* p. H63D，c.1006+8G＞A | 37 | M | 1316 | 85 | 19 | 15 | 21 | 10 | 18 | Liver | ND | Abnormal liver function test |
| 17 | *HJV* p. Q6H/ C321X | 30 | M | 11555 | 98 | 103 | 77 | 103 | 14 | 5 | Liver, spleen, and pancreas | ND | Cardiac failure, diabetes, loss of libido and skin pigmentation |
| 18 | *HJV* p. Q6H(homo)/ C321X(homo) | 24 | M | 6037 | 94 | 61 | 55 | 19 | 4 | 49 | Liver, pancreas and heart | ND | Cardiac failure, cardiopathy, atrial fibrillation, abnormal liver function test, diabetes, hypothyroidism |

ALT, alanine aminotransferase; AST, aspartate aminotransferase; ND: Not done; SF, serum ferritin; T-Bil, total bilirubin; D-Bil, direct bilirubin; TS, transferrin saturation; γ‐GGT, gamma glutamyl transpeptidase; MRI, magnetic resonance imaging
