# Supplementary material for: Identification of novel non-HFE mutations in Chinese patients with hereditary hemochromatosis
Source: Orphanet J Rare Dis. 2022 Jun 6;17:216. doi: 10.1186/s13023-022-02349-y (PMC9169345; doi:10.1186/s13023-022-02349-y)
Supplement: Supplementary file 2 — Additional file 2. Table S2. Primers for the Sanger sequencing of UBE2O, PCSK-7 gene. [file 13023_2022_2349_MOESM2_ESM.docx]

Table S2. Primers for the Sanger sequencing of *UBE2O* and *PCSK-7* gene

| Gene | Exons | Forward | Reverse | Length  （bp） | Annealing temperature (°C) |
| --- | --- | --- | --- | --- | --- |
| ***UBE2O*** | Exon 1 | CACGCAGGGACACGTCA | TACCCTCCACTGGGGCTG | 677 | 60 |
|  | Exon 2-3 | CAGTGTGGATGTGACGCTTT | GATGATGAGAAGACAGGGCCG | 492 | 60 |
|  | Exon 4-5 | GTCTTCGGTGGGGCATAGAG | TGGGTCCAGGTCTTTGCTAC | 806 | 60 |
|  | Exon 6 | CTTTCCTTTGGCCCCACATCC | CAACCCTCAAGAGCAGGAAGG | 263 | 60 |
|  | Exon 7-8 | CTGGCTAAAGAGAGTGCTGTCA | TCCTCAAATGCCCACCAATC | 505 | 60 |
|  | Exon9 | CTCACAGCCCTGTCCTCTCT | CTTCACGCTGACGCCATTG | 552 | 60 |
|  | Exon10 | GGGACAGGAAGGGACAGAAAGAC | GATCCACTGCCCATTCTCCACAA | 326 | 60 |
|  | Exon11-12 | GCCGGGATCCCTTAGCTAGT | AGCAGCCATCCAGAACTTGAA | 409 | 60 |
|  | Exon 15 | GCCTCTCCATGAGGGACTGA | CACACAGCACATCTGCACCT | 219 | 60 |
|  | Exon 16-17 | GTCCTGGGAAAGAGCAGGTG | CACAATGCAGGCCTACCCTC | 555 | 60 |
|  | Exon 18 | TGGAGGGTAGGCCTGCATTG | CTCTCCCACGGTGATGCTCTT | 773 | 60 |
| ***PCSK7*** | Exon1 | TAGGGACTTCCAGGGGGAAC | ACTGGAACCTACCCACCTGA | 632 |  |
|  | Exon2 | ATCTGTCTGGCTCTTTGGTGG | GGAGTGAGGGGTGTCGTTC | 281 |  |
|  | Exon3 | ATGACCACATGTCACCTCCA | TGTTTCAGAGTAGGGAGGGGT | 282 |  |
|  | Exon4 | ATTAGTGAGGAGCTGCTGTGG | CCTAAGTCAGCCCACTGGTT | 215 |  |
|  | Exon5 | CTGGCTGTCCCCTCTAGGTA | AGTCTTTGTGCAGGCCAGTT | 112 |  |
|  | Exon6 | GGGTTCCTCAACTGACACCC | AGGACAAGACGTGTGATGCC | 249 |  |
|  | Exon7 | CCCCCAGGATGGTAACTCCA | AACAGTGAGAAGCGCTGTGA | 280 |  |
|  | Exon8 | TCCTTTTCCTGTTTCACTTCCAG | TAAGGCCACCTGATACCCGA | 261 |  |
|  | Exon9 | AAGCAGGGAAACCAGAGTCAG | ACCCTTGGTCACTCCACACA | 235 |  |
|  | Exon10 | TATTGACAAGGGCGTGTGTGT | CGAAAGGGGGTAGAATCTGGC | 291 |  |
|  | Exon11 | GTGTTTGTGGGCTGGTTTCC | GGCCCTTGCTGAAAAGGTTG | 249 |  |
|  | Exon12 | ACCCTCCCAGGTTGTTTCCA | GCACACGGGACACTTTCTGT | 297 |  |
|  | Exon13 | CTCCTGGAGCAGACCATGTG | TTGACGCTGGAACTGGGAAG | 319 |  |
|  | Exon14 | AAATGGGTGAGGGCAGATGG | TACCCGCAGCTTGAGTCTTG | 218 |  |
|  | Exon15 | GGGAAACAGTGCCTCACGTA | GAAGAGCCTGTCCCACACTG | 433 |  |
