# Supplementary material for: Identification of novel non-HFE mutations in Chinese patients with hereditary hemochromatosis
Source: Orphanet J Rare Dis. 2022 Jun 6;17:216. doi: 10.1186/s13023-022-02349-y (PMC9169345; doi:10.1186/s13023-022-02349-y)
Supplement: Supplementary file 3 — Additional file 3. Table S3. Quality control of the whole exome sequence of the 9 cases with primary iron overload. [file 13023_2022_2349_MOESM3_ESM.docx]

Table S3 Quality control of the whole exome sequence of the 9 cases with primary iron overload

| Sample | RawData(Mb) | CleanData(Mb) | Mapped target | Coverage (%) | Depth | ≥4×（%） | ≥10×（%） | ≥20×（%） | Duplication（%） |
| --- | --- | --- | --- | --- | --- | --- | --- | --- | --- |
| 18C059512 | 9871.83 | 9537.39 | 9515.45 | 99.74 | 77.24 | 98.48 | 98.48 | 94.87 | 20.88 |
| 19C035297 | 11755.5 | 11403.95 | 11376.7 | 99.91 | 99.56 | 99.56 | 98.63 | 95.34 | 22.84 |
| 19C035236 | 9540.65 | 9457.5 | 9430.02 | 99.71 | 59.56 | 97.2 | 97.20 | 88.57 | 23.53 |
| 18C065854 | 13132.15 | 12894.18 | 12866.68 | 99.92 | 95.09 | 99.66 | 98.97 | 96.75 | 19.20 |
| 19C035302 | 10687.7 | 10542.49 | 10521.66 | 99.94 | 79.67 | 99.71 | 98.96 | 95.97 | 16.13 |
| 19C035292 | 9384.95 | 9071.18 | 9042.18 | 99.94 | 72.24 | 99.64 | 98.60 | 94.46 | 20.27 |
| 19C035291 | 10358.05 | 9796.56 | 9742.46 | 99.94 | 75.09 | 99.65 | 98.62 | 94.58 | 18.22 |
| 19C035298 | 9297.86 | 8881.66 | 8861.24 | 99.92 | 69.42 | 99.56 | 98.29 | 93.43 | 20.08 |
| 18C065856 | 12102.67 | 11744.8 | 11720.56 | 99.92 | 90.51 | 99.63 | 98.87 | 96.26 | 19.25 |
