# Supplementary material for: Identification of novel non-HFE mutations in Chinese patients with hereditary hemochromatosis
Source: Orphanet J Rare Dis. 2022 Jun 6;17:216. doi: 10.1186/s13023-022-02349-y (PMC9169345; doi:10.1186/s13023-022-02349-y)
Supplement: Supplementary file 4 — Additional file 4. Mutations identified in known iron metabolism–related genes or genes associated with iron metabolism pathway by NGWES. [file 13023_2022_2349_MOESM4_ESM.docx]

Table S4. Missense variants identified in genes associated with iron metabolism pathway genes identified in patients with primary iron overload by the whole exome sequencing

|  |  |  |  |  | Polyphen-2 | |  | SIFT | |  | Mutation Taster | |
| --- | --- | --- | --- | --- | --- | --- | --- | --- | --- | --- | --- | --- |
| Gene (accession number) | Amino acid change | Base change | ExAC | gnomAD_exome | Prediction | Score |  | Prediction | Score |  | Prediction | Score |
| *UBE2O* | p.K689R | c.2066A>G | 0.0027 | 0.0028 | Probably damaging | 0.993 |  | Tolerable | 0.853 |  | Disease causing | 1 |
| *PCSK7* | p.S550R | c.1650T>A | - | - | Probably damaging | 0.997 |  | Damaging | 0.032 |  | Disease causing | 1 |
| *PCSK7* | p.R711W | c.2131C>T | 0.0011 | 0.0012 | Probably damaging | 0.978 |  | Damaging | 0.005 |  | Polymorphism | 1 |
| HEPH | p.T79I | c.236C>T | - | - | Benign | 0.005 |  | Tolerated | 0.294 |  | Disease_causing | 1 |
| ADAMTS7 | p.G105S | c.313G>A | 0.0003 | 0.0003 | Probably_damaging | 0.999 |  | Tolerated | 0.209 |  | Disease_causing | 1 |
| ADAMTS7 | p.G1587S | c.4759G>A | 0.001 | 0.0006 | Probably_damaging | 0.998 |  | Tolerated | 0.094 |  | Disease_causing | 1 |
| POLE | p.V1514A | c.4541T>C | 0.0002 | 0.0002 | Benign | 0.168 |  | Tolerated | 0.148 |  | Disease_causing | 1 |
| NUBPL | p.S82Ifs*67 | c.240dupA | - | - | - | - |  | - | - |  | - | - |
| NAT2 | p.E265X | c.793G>T | - | - | - | - |  | - | - |  | Disease_causing | 1 |
| CPOX | p.Q29R | c.86A>G | - | - | Benign | 0 |  | Damaging | 0.024 |  | Polymorphism | 1 |
| BTN1A1 | p.F4L | c.12C>A | 0.00001648 | 0.00001624 | Benign | 0.001 |  | Damaging | 0.03 |  | Polymorphism | 1 |
| ALAD | p.V165I | c.493G>A | - | - | Benign | 0.024 |  | Tolerated | 0.302 |  | Disease_causing | 1 |
| CYP1A1 | p.Q75P | c.224A>C | 0.0001 | 0.0001 | Probably_damaging | 0.983 |  | Damaging | 0.001 |  | Disease_causing | 1 |
| VSIR | p.N224H | c.670A>C | - | - | Benign | 0.001 |  | Tolerated | 1 |  | Disease_causing | 1 |
| BTBD9 | p.L55M | c.163T>A | - | 0.000004064 | Probably_damaging | 0.999 |  | Damaging | 0.003 |  | Disease_causing | 1 |
| CCND1 | p.X296delinsX | c.888_889insGGGCGCCAGGCAGGC | - | - | - | - |  | - | - |  | - | - |
| NDUFV1 | p.L35V | c.103C>G | - | - | Possibly_damaging | 0.652 |  | Damaging | 0.007 |  | Disease_causing | 1 |
| CTDSPL2 | p.T268A | c.802A>G | - | - | Benign | 0.122 |  | Tolerated | 0.211 |  | Disease_causing | 1 |
| HEYL | p.R233W | c.697C>T | 0.00005386 | 0.00004341 | Possibly_damaging | 0.549 |  | Damaging | 0.001 |  | Disease_causing | 0.723 |
| BMPR1B | p.R26P | c.77G>C | 0.00003303 | 0.00001627 | Benign | 0 |  | Tolerated | 0.394 |  | Disease_causing | 0.691 |
| NOTCH1 | p.E2254K | c.6760G>A | - | 0.000004212 | Benign | 0.23 |  | Damaging | 0.045 |  | Disease_causing | 1 |
| ACO2 | p.R767H | c.2300G>A | 0.0001 | 0.0002 | Benign | 0.005 |  | Damaging | 0.008 |  | Disease_causing | 1 |
| RUNX2 | p.78delA | c.232_234delGCG | 0.0006 | 0.0023 | - | - |  | - | - |  | - | - |
| SKOR1 | p.751_752delSGinsS | c.2252_2254delGCG | 0.0184 | 0.0047 | - | - |  | - | - |  | - | - |
| FBN1 | p.V266I | c.796G>A | 0.00001648 | 0.000008122 | Possibly_damaging | 0.527 |  | Tolerated | 0.343 |  | Disease_causing | 1 |
| GATA6 | p.P54A | c.160C>G | 0.00001095 | 0.00001383 | Benign | 0.018 |  | Damaging | 0.031 |  | Polymorphism | 0.875 |
| RNF165 | p.G146D | c.437G>A | - | - | Benign | 0.066 |  | Damaging | 0.004 |  | Disease_causing | 1 |
| RTEL1 | p.92delA | c.274_276delGCT | 0.003 | 0.0003 | - | - |  | - | - |  | - | - |
| CARMIL1 | p.K1066R | c.3197A>G | - | - | Benign | 0.009 |  | Tolerated | 0.229 |  | Disease_causing | 0.792 |
| LRP4 | p.R1361C | c.4081C>T | 0.000008237 | 0.0000122 | Probably_damaging | 0.972 |  | Damaging | 0 |  | Disease_causing | 1 |
| LPA | p.1972_1974delKYIinsI | c.5915_5920delAGTATA | 0.0001 | 0.00009348 | - | - |  | - | - |  | - | - |
| REV3L | p.R3073Q | c.9218G>A | 0.00001647 | 0.00001625 | Possibly_damaging | 0.667 |  | Damaging | 0.041 |  | Disease_causing | 0.999 |
| GDF7 | p.G41delinsGGG | c.123_124insGGCGGC | - | 0 | - | - |  | - | - |  | - | - |
| FN1 | p.V1139I | c.3415G>A | - | 0.0000325 | Probably_damaging | 0.997 |  | Tolerated | 0.161 |  | Polymorphism | 0.993 |
| USP9X | p.F285L | c.855T>G | - | - | Benign | 0.001 |  | Tolerated | 1 |  | Disease_causing | 1 |
| MEGF8 | p.R1142W | c.3424C>T | 0.0011 | 0.0008 | Possibly_damaging | 0.82 |  | Damaging | 0.003 |  | Disease_causing | 0.958 |
| SOSTDC1 | p.S198Afs*7 | c.592delA | 0.0036 | 0.004 | - | - |  | - | - |  | - | - |
| ABCG2 | p.360_361delKIinsI | c.1079_1081delAGA | 0.0001 | 0.0001 | - | - |  | - | - |  | - | - |
| ARSB | p.30delL | c.88_90delCTG | 0.0016 | 0.001 | - | - |  | - | - |  | - | - |
| IREB2 | p.M102V | c.304A>G | 0.0003 | 0.0004 | Probably_damaging | 0.956 |  | Tolerated | 0.195 |  | Disease_causing | 1 |
| SLC22A17 | p.M154V | c.460A>G | 0.0005 | 0.0006 | Benign | 0.33 |  | Damaging | 0.047 |  | Disease_causing | 0.998 |
| WDR75 | p.F746S | c.2237T>C | 0.0013 | 0.0012 | Possibly_damaging | 0.563 |  | Tolerated | 0.25 |  | Disease_causing | 0.968 |
